# Cultural barriers to stunting prevention: a case study of the Baduy indigenous tribe in Indonesia

**DOI:** 10.3389/fsoc.2026.1724639

**Published:** 2026-03-04

**Authors:** Yustia Atsanatrilova Adi, Sri Hilmi Pujihartati, Rahesli Humsona, Dyke Gita Wirasisya

**Affiliations:** 1 Department of Sociology, Faculty of Social and Political Sciences, Universitas Sebelas Maret, Surakarta, Indonesia; 2 Institute of Pharmacognosy, University of Szeged, Szeged, Hungary

**Keywords:** Bourdieu framework, cultural capital, habitus, indigenous health, symbolic violence

## Abstract

Stunting is widely understood as a chronic manifestation of malnutrition. In Indonesia, the national prevalence of stunting in 2024 stands at 19.8%; however, in the Baduy tribe of Banten Province, stunting prevalence have been reported as high as 60%. This disparity raises a critical question: what cultural barriers significantly hinder stunting prevention within the Baduy community? To address this question, we conducted the present study that employed a qualitative research design with purposive sampling, drawing on in-depth data from 20 informants representing both Baduy Dalam and Baduy Luar tribes. From this study, we identify three dominant cultural barriers related to stunting: limited nutritional knowledge which resulting from restrictions on formal schooling, strict prohibitions against keeping or slaughtering four-legged animals which limit their nutritional intake, and constraints on the use of modern transportation. This study also demonstrates that processes of embodiment, objectification, and institutionalization in the development of cultural capital are effectively sustained within Baduy society; however, these processes also simultaneously reinforce social separation from the broader population and consolidate a traditional belief system that poses significant challenges to public health interventions. Addressing stunting in this context therefore requires culturally embedded strategies that institutionalize collaboration between traditional leaders (*jaro*) and health workers (*bidan*). Such strategies should be framed in modern health practices through locally meaningful symbols, ensuring alignment with Indonesia’s legal framework for child protection and stunting reduction, as articulated in Presidential Regulation No. 72 of 2021, Law No. 35 of 2014 on Child Protection, and Law No. 17 of 2023 on Health.

## 1 Introduction

*Stunting* is a condition where children are considered to be too short compared with their age because of recurrent or chronic malnutrition (WHO, 2015). This condition is mostly caused by the lack of nutrients consumed by the mother during pregnancy, as well as a lack of nutrition provided to the child during the first 2 years of life (Saleh et al., 2021). According to several studies, *stunting* can be caused by various essential variables, such as family characteristics, infectious disease (mostly caused by a lack of immunization program), and structural factors (poverty, less educated parents, etc.) (Danso and Appiah, 2023; Ismawati et al., 2020; Prendergast and Humphrey, 2014). *Stunting* in early childhood impairs physical growth and is associated with adverse functional impact, including below-average stature and delayed motor development. In the long term, *stunting* is further linked to diminished cognitive capacity and an increased risk of nutrition-related chronic diseases in adulthood (de Onis and Branca, 2016). In the wider perspective, *stunting* also perpetuates intergenerational cycles of poverty and compromises both individual life trajectories and national economic development (Prendergast and Humphrey, 2014). Stunting is also responsible for half of the fatalities of children under the age of five in low and middle-income nations (WHO, 2024). Therefore, stunting prevention is necessary since early abnormalities in brain development and skeletal growth cannot be recovered.

In 2024, 150 million, or 23.2% of the child population worldwide, were identified as stunted. To reduce the number of stunting, the World Health Organization (WHO), United Nations Children’s Fund (UNICEF), and the World Bank are collaborating to prevent stunting by launching the WASH program, which includes interventions for food, health, water, sanitation, and hygiene, social protection, and education (for maternal nutrition). In general, from 2021 to 2024, the WASH program has demonstrated encouraging improvement, with the global prevalence of stunting declining from 24.4% to 23.2% (WHO, 2024). However, the trends do not reflect the global picture of stunting reduction, whereas, in some countries, such as Burundi, the stunting rate has remained stable at 56.6% from 2019 to 2023, owing to structural poverty, which is highly correlated with the mother’s ability to obtain nutrition for their newborn (Gaiser et al., 2023).

On the other hand, positive development in the stunting prevention program is emerging in Southeast Asia, with stunting decreasing by around 1.5% each year beginning in 2021, with the most recent figure reaching 27.4% (Azriani et al., 2024). As one of the Southeast Asian countries, Indonesia also shows positive trends in stunting prevention. In 2021, stunting prevalence remained high at 31.8% but declined substantially to 19.8% by 2024 (Ministry of Health, 2025). However, the province of Banten, located less than 100 kilometres from the capital city, Jakarta, continues to face persistent challenges related to child stunting. According to the 2022 *Indonesian Nutrition Status Survey* (SSGI), the provincial stunting prevalence was reported at approximately 21.6% (Kemenkes RI, 2022). Furthermore, significant disparities exist within the region, whereas the stunting prevalence among children in the Baduy tribes community, based on the research conducted in 2024, reached up to 60% (Putri et al., 2024). This figure is notably higher than the surrounding regencies and cities, such as Tangerang City and South Tangerang City, where stunting prevalence has fallen well below the national average, at approximately 11.8% and 9.0%, respectively (Kemenkes RI, 2022).

This condition is aggravated by the fact that the research on the Baduy remains significantly limited. Recent studies have shown the absence of comprehensive cultural factors that could explain the barriers to successful health interventions, whereas most studies have primarily focused on health perspectives. One of the recent studies, conducted by Ritanti et al. (2025), examined healthcare infrastructure gaps, such as the lack of a community health centre (Posyandu) in the Baduy Area (Ritanti et al., 2025). Meanwhile, Putri et al. (2024) assessed socioeconomic factors requiring government support (Putri et al., 2024). The recent research, which was conducted by Rohmatullayaly et al. (2025), using the public health perspective, by analysing the data from the community health center (*Posyandu*), found that the Baduy children aged 0–5 years reveal a unique growth pattern characterized by small body size and slow growth rate (Rohmatullayaly et al., 2025).

The literature assessments above identify a large study vacuum addressing cultural barriers to stunting prevention in the Baduy tribes. This gap becomes particularly critical when considering Indonesia’s persistent struggle with stunting, where, despite national efforts to achieve a 14% prevalence reduction by 2030, the country’s *Global Hunger Index* score is 16.9%, reflecting moderate levels of hunger, with rural and indigenous communities disproportionately affected (Tarmizi, 2024). Therefore, this research represents the first comprehensive sociological study to apply Bourdieu’s theoretical framework to examine the previously unexplored relationship between cultural capital, habitus formation, and health intervention resistance that has been entirely overlooked in the existing literature on this critically understudied community.

## 2 Literature review

### 2.1 Global and national burden of stunting and structural determinants

**Figure 1:**
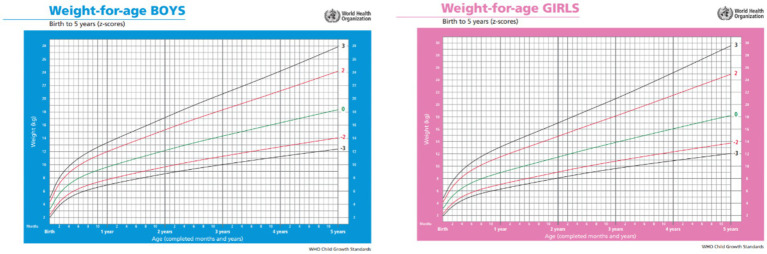
WHO children growth standard. Source: WHO (2009).

As mentioned in the background section, *stunting* remains a primary global health challenge, affecting approximately 148 million children under the age of five, or 23.2% of the total population of children worldwide in the year 2024 (WHO, 2024). Based on *the Stunting Screening Indicator* (SSI) which shown in Figure 1, the WHO is defines normal growth in children under five as having both a height-for-age z-score (HAZ) and a weight-for-age z-score (WAZ) between −2 and +2, which on the WHO growth charts roughly translates to a 1-year-old measuring about 75 cm (±2.5 cm) and weighing 9.5 kg (±1.1 kg), a 2-year-old at 87 cm (±2.8 cm) and 12.2 kg (±1.3 kg), a 3-year-old at 95 cm (±3.0 cm) and 14.2 kg (±1.5 kg), a 4-year-old at 102 cm (±3.2 cm) and 16.0 kg (±1.7 kg), and a 5-year-old at 109 cm (±3.4 cm) and 18.0 kg (±1.9 kg). Any child whose HAZ falls below 2 is considered *stunted* and requires closer attention (WHO, 2009).

According to a recent research, *stunting* occurs as a result of complex structural determinants such as the poverty rate of a country or region, malnutrition of pregnant women and newborn babies, and limited access to healthcare services, which create complex layers of vulnerability within the stunting prevention program (Mulatu et al., 2025). Similarly, a related study conducted in Nepal has found that in low and middle-income countries, stunting prevalence can often be attributed to maternal health, suboptimal breastfeeding practices, and inadequate complementary feeding (Tiwari et al., 2014). However, the burden of stunting seems not uniformly distributed. In regions like Ethiopia and Tanzania, environmental and sanitation determinants play a crucial role. Kwami et al. (2019) assert that poor water, sanitation, and hygiene conditions severely limit childhood nutritional outcomes (Kwami et al., 2019). This observation is supported by Chirande et al. (2015), who found that a significant portion of child mortality and disease burden can be traced back to maternal and childhood undernutrition, including stunting, in developing contexts (Chirande et al., 2015). These studies show the relationship between environmental health variables and physiological causes of stunting, suggesting a comprehensive framework for integrated health and nutrition policies. Later, as relevant according to previous findings, improvements in sanitation and maternal health services have a positive correlation with enhanced child growth outcomes, underscoring the necessity for a comprehensive approach that considers both immediate health determinants and broader environmental influences on child development (Vaivada et al., 2020).

Within Indonesia, the existing studies suggest that ineffective implementation of stunting prevention policies, such as budget constraints and inadequate staffing, has resulted in suboptimal outcomes in remote areas, such as Nagari Pulakek Koto Baru, South Solok Regency, Indonesia (Milwan and Sunarya, 2023). The finding was also verified by a qualitative study conducted by Prastiwi et al. (2020), which concluded the importance of sanitation and health education, underlining that poor living conditions, including a lack of clean water, worsen the incidence of stunting among indigenous children (Prastiwi et al., 2020).

Specifically, in relation to the *stunting* eradication in the indigenous group, based on a study conducted by Putri et al. (2025), as observed in the Baduy community, the hereditary tendency is believed to be the primary cause of stunting, rather than a result of environmental and nutritional deficiencies (Putri et al., 2025). These phenomena also happened in a different region, such as the Chiapas highland, Mexico, as research conducted by Gutiérrez-Jiménez et al. (2019) found that the high rates of stunting in marginalized groups are connected with restricted access to vital health services (Gutiérrez-Jiménez et al., 2019). Moreover, as related to the cultural barrier, Sinaga et al. (2023) found that cultural differences hinder community engagement in government health activities, leading to inadequate knowledge and use of available resources for stunting prevention (Sinaga et al., 2023). It can be concluded, based on numerous previous studies, that resolving stunting in indigenous communities necessitates a diverse approach that must prioritize cultural sensitivity, as the indigenous group may have various approaches to the specific policy.

### 2.2 Baduy tribes cultural practices, health, illiteracy and nutrition behaviors

This research focused primarily on the Baduy tribes as one of the indigenous populations in Indonesia, which is known for its strong commitment to traditional norms. Baduy, who mainly reside in West Java, Indonesia, follow very strict dietary guidelines based on their cultural knowledge, which significantly influence their nutritional intake and health practices. This statement is supported by one of the limited studies available, conducted by Sukandar and Mudjajanto (2012), who found that the Baduy people maintain their diet according to the cultural knowledge that has been handed down through generations, which mainly relies on locally derived foods (Sukandar and Mudjajanto, 2012). However, as confirmed by Rohmatullayaly et al. (2025), such traditional practices can foster health misinformation and limit the community’s exposure to modern nutritional information, reflecting a culturally rooted form of health illiteracy (Rohmatullayaly et al., 2025).

The concept of health literacy in Baduy may differ from modern ones. Health literacy itself can be defined as an individual’s ability to understand and use health knowledge in a culturally relevant environment and translate it into a health-related action (Liu et al., 2020). Furthermore, restricted interactions with the existing health systems might be a key obstacle in the function to communicate critical health information, resulting in a health disparity (Jindal et al., 2023). The concept above is further supported by a study conducted by Chao and Kang (2020), which focuses on Bhutanese adult refugees and found the usefulness of culturally adapted interventions to promote health literacy and could be relevant in a broader sense (Chao and Kang, 2020). In the broader context of public health, the implications of low health literacy in indigenous communities emphasize the need for a specific approach. Farmanova et al. (2018) research on organizational health literacy in a specific community has a favorable impact on healthcare outcomes; thus, it is pertinent to the subject of improving health communication in the Baduy setting. Given this, it is critical to recognize the Baduy’s distinct cultural context and historical practices. The interplay of cultural practices, health literacy, and nutritional behaviors within the Baduy indigenous community presents complex challenges that require in-depth investigation to improve health literacy among the Baduy people.

### 2.3 Absence of cultural barrier analysis: the identified research gap

Furthermore, rather than being viewed as obstacles with structure, cultural beliefs are often relegated to background descriptions. For instance, there are national health strategies conducted by the Indonesian government, such as *Gerakan Nasional Percepatan Perbaikan Gizi,* or *National Movement for Accelerated Nutrition Improvement*. This intervention from the community health center *(Posyandu)* aims to reduce the number of *stunted children* (Sunaryo et al., 2023). However, what happens is the opposite; based on a survey conducted by the Ministry of Health on the Baduy family, it showed that only 22% of respondents received information from health workers or midwives; most of them still rely on neighbors or relatives. Customary leaders accounted for only 7% of health information channels, which can be an index of the misalignment between official channels and trusted authority structures (Kemenkes RI, 2022). From our analysis in the literature review part, numerous items can be concluded once the research gap is closed, as indicated below:

- *Empirical gap*: according to our assessment, no research has comprehensively examined the cultural beliefs that are also considered active impediments to stunting intervention in the Baduy indigenous community.
- *Conceptual gap*: based on prior studies conducted in the Baduy community, culture-related factors are only viewed as a tangential element rather than an organized system defining the stunting prevention program. Norms, taboos, fatalism, and leadership impact have all received little attention.

Based on our extensive literature review, this study is the first to completely investigate cultural belief systems and norms as key impediments to eradicating stunting in the Baduy indigenous group. This study’s findings are critical for developing culturally appropriate, community-based stunting treatment options that respect identity, employ trusted agents, and arise through discourse rather than imposition.

## 3 Methods

### 3.1 Research design

**Figure 2:**
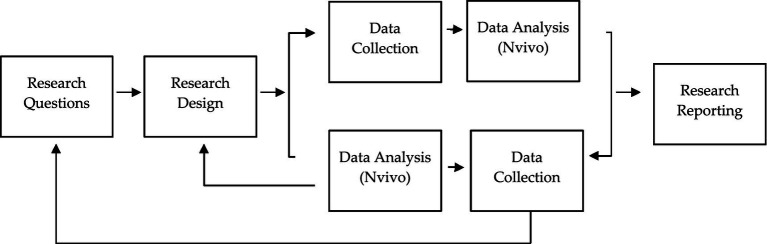
Research framework.

This study employs a qualitative descriptive design as its primary research framework. Given that qualitative research is characterized by flexibility, openness, and responsiveness to context, the procedures of data collection and analysis are not as strict as they are in quantitative research, which is needed for the exploration of the indigenous community (Lim, 2024). The data is analysed by the *interpretive-constructivist* paradigm to deeply investigate the cultural factors impacting the ongoing stunting prevalence among the Baduy indigenous community in Banten province, Indonesia, particularly concerning nutrition and child growth. Furthermore, the decision to use a qualitative approach is justified by the lack of prior cultural investigations in the Baduy context, the limitation of the available numerical data specific to indigenous subpopulations, and in relevance to the aim of the study, to capture contextualized lived experiences through emic perspectives (Creswell, 1998). To summarize, this research was conducted using the following research logic (Figure 2):

The research framework illustrated in the image represents a step-by-step guide which used in this research. The procedure begins with the collection of the prior research that has previously been completed regarding the Baduy. The findings of interviews, observations, and associated documents are gathered (Tarnoki and Puentes, 2019). This data is analysed to identify patterns, meanings, and insights using the Nvivo program, and the open nodes classification is utilized to optimize the findings from the interview transcript (Feng and Behar-Horenstein, 2019). Importantly, the results of this analysis, which have already been collected by the Nvivo program, are not final; rather, they feed back into the research phase, altering questions and leading to more data gathering to maximise the depth of the interview guide (Dhakal, 2022). This dynamic movement between interview data, analysis, and theoretical analysis mirrored the non-linear, reflexive, and emergent nature of qualitative investigation (Jennings, 2012). Finally, in the research reporting phase, the final version of the analytical results is analyzed using grounded theory to highlight constant comparison, theoretical sampling, and the co-construction of the research findings (Urcia, 2021). Thus, rather than following a fixed linear path, qualitative research unfolds dynamically through cycles of reflection and adaptation.

### 3.2 Study location and context

**Figure 3:**
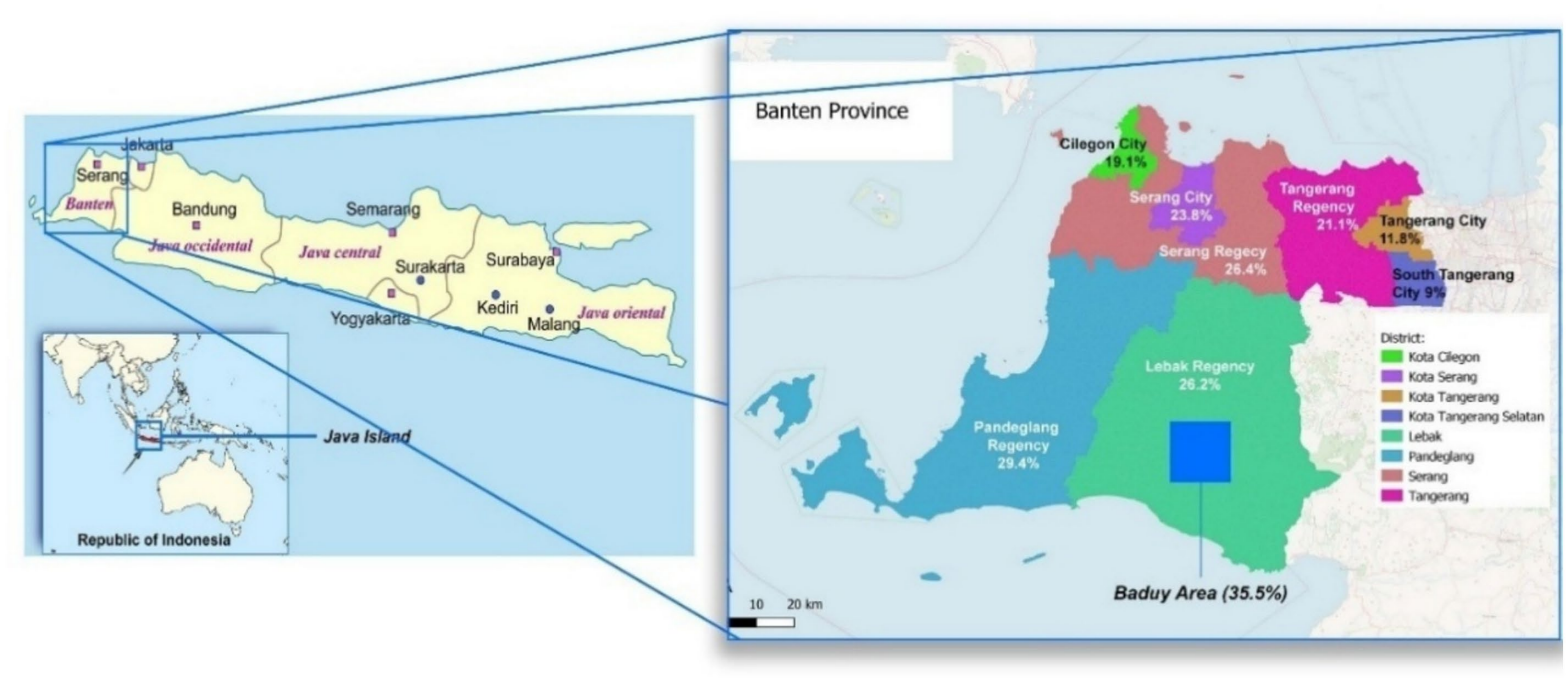
The research location within the Indonesian map, and the stunting percentage based on SSGI 2022.

**Figure 4:**
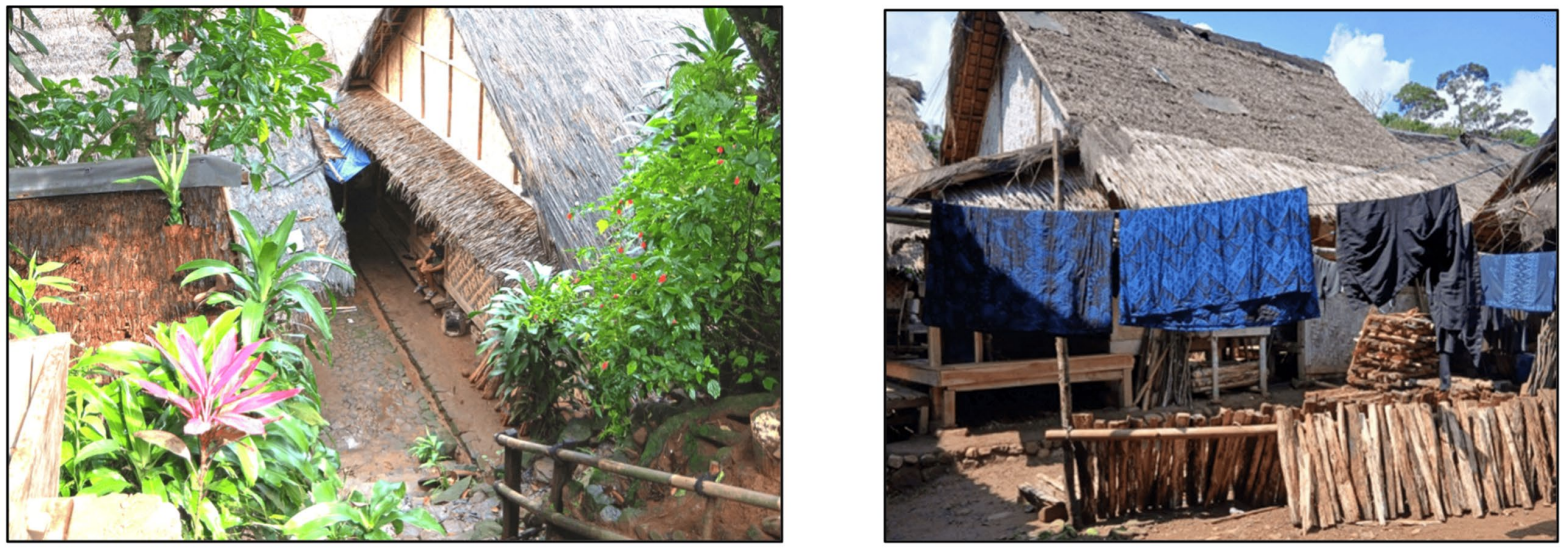
The image of the Baduy indigenous community house who residing in Kanekes Village, Banten, Indonesia, approximately 5 h of walking from the nearest public road deep to the forest. Source: Authors.

This research was conducted in the Kanekes Village, Lebak Regency, Banten Province, where the Baduy Indigenous community resides. The research region is physically close to Indonesia’s capital (Jakarta), yet there was a substantial socio-cultural distance between Kanekeas village and Jakarta due to a long-standing rejection of modernization, including official healthcare, education, and infrastructure development (Utami et al., 2023). In 2022, the Lebak regency reported a stunting rate of 35.5% (Kemenkes RI, 2022), Based on the latest numerical results, it was estimated that stunting prevalence in the Baduy indigenous community exceeds 60%, the highest in Banten Province (Putri et al., 2025). According to field observations, the village is served by nine community health centres *(Posyandu)* that are unfortunately unable to function properly due to resistance or non-cooperation from residents. These inconsistencies and sociocultural resistances make the location ideal for evaluating how cultural barriers posed significant challenges to stunting prevention (Figures 3, 4).

### 3.3 Participant selection

We used a *purposive sampling* method in this study to maximize the variation criteria to ensure representation across gender, age, social role, and involvement in healthcare or caregiving, which is widely distributed, so that it can represent the Baduy indigenous community that lives in the Baduy area (Campbell et al., 2020). A total of 20 informants from different backgrounds were interviewed (Table 1):

**Table 1:** Informant datasheet.

Informant name (initial)	Age classification	Marital status	Number of children	Occupation	Sex
AL	Adult (25–45 Years)	Married	1	SME Trader	Female
AS	Youth (15–24 Years)	Married	1	Farmer	Male
DD	Youth (15–24 Years)	Married	1	SME Trader	Male
AN	Youth (15–24 Years)	Married	2	Farmer	Male
AH	Adult (25–45 Years)	Married	2	Farmer	Male
SK	Adult (25–45 Years)	Married	2	Housewife	Female
NE	Adult (25–45 Years)	Married	1	SME Trader	Female
NI	Adult (25–45 Years)	Married	2	Housewife	Female
PUS	Adult (25–45 Years)	Married	1	Civil Servant	Male
JAR	Youth (15–24 Years)	Unmarried	0	Farmer	Male
MER	Adolescents (10–14 Years)	Unmarried	0	Not Applicable	Female
ER	Senior (45 Above)	Married	2	Midwife	Female
GIU	Adult (25–45 Years)	Married	0	Farmer	Male
SHA	Senior (45 Above)	Married	2	Shaman	Male
VS	Senior (45 Above)	Married	1	Civil Servant	Male
NIS	Youth (15–24 Years)	Married	1	Housewife	Female
JRO	Adult (25–45 Years)	Married	2	Housewife	Female
JM	Youth (15–24 Years)	Married	1	Farmer	Male
FP	Adult (25–45 Years)	Married	0	Midwife	Female
OD	Youth (15–24 Years)	Married	2	SME Trader	Female

The selected participants were identified through strict coordination with local *Posyandu* personnel, head of the tribes, and village gatekeepers, consistent with protocols for research in indigenous or guarded communities. Before beginning the data collection, the authors made sure that the key person in the villages was already informed and had agreed (Williams and Shipley, 2023). Before interviews, verbal consent was obtained in accordance with ethical research standards for oral and low-literacy communities.

### 3.4 Data collection procedures

Data were gathered between April and June 2025 through semi-structured interviews aiming to elicit perceptions, beliefs, and behaviors about child health, stunting, and health services. Furthermore, to keep the answers on track and consistent with the research aims, the field interviews were led by six major themes, which aligned based on the previous research, those are:

1. Cultural interpretations of child growth and health
2. Daily food practices and nutrition taboos
3. Pregnancy and postnatal rituals
4. Traditional health remedies and disease interpretation
5. Experiences with public health programs
6. Community trust, leadership, and decision-making in health

All interviews were conducted in *Bahasa Indonesia* and Bahasa Sunda *(Sundanese)* using a mixture of standard and informal dialects. When required, local bilingual assistants supported clarification. Interview durations ranged from 30 to 60 min. Conversations were recorded with permission and supplemented by field notes to capture environmental cues, body language, and community dynamics. The interview guide was pilot-tested with one non-sample participant and adjusted to improve cultural appropriateness and question flow. The finalized guide is presented in Table 2.

**Table 2:** Semi-structured interview guide.

Domain	Key questions
Cultural beliefs about child health	How do you and your community understand what makes a child healthy? What is the community’s view of children who are small or short? Are there specific terms or beliefs used to explain stunting or short growth?
Daily food practices and nutrition	What foods do you usually prepare for your children? Are any foods avoided during pregnancy, breastfeeding, or childhood? How often do children eat each day? Is food availability ever a problem?
Maternal and child health practices	What do mothers usually do after giving birth? Do pregnant women or mothers attend Posyandu or visit midwives? Are there traditional postnatal rituals or practices?
Health-seeking behavior	What do families do when a child gets sick? What traditional herbs or healing practices are used? Have you visited a health facility (Puskesmas or hospital)? Why or why not?
Experience with health programs	Have health workers or Posyandu staff visited your area? What do you think about programs like food supplementation or child growth monitoring? What makes people accept or reject these services?
Community norms and trust	Who do you trust most for health-related advice—health workers, elders, or traditional healers? Are there taboos or prohibitions against using health services? Do customary leaders influence health decisions?

**Figure 5:**
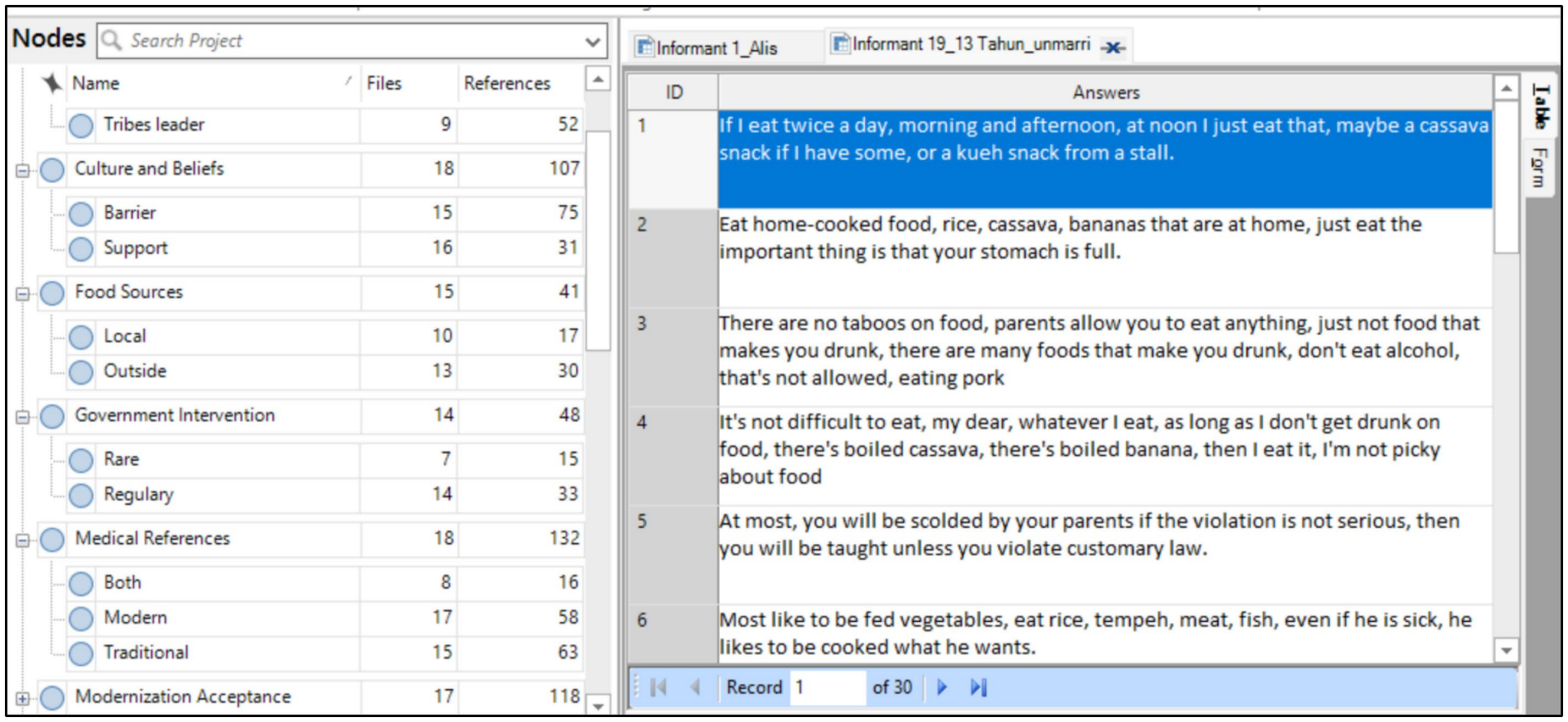
The process of analysing interview results using the open nodes method in NVivo 12 Pro software.

All transcribed interview data were imported into the NVivo 12 Plus software for systematic thematic analysis. The six-step process, which was introduced by Braun and Clarke (2006), was followed, as explained as follows (Braun and Clarke, 2006):

1. *Familiarization:* Transcripts were read multiple times, and notes were taken.
2. *Initial Coding:* Open coding was conducted inductively to stay close to the data, avoiding pre-established categories.
3. *Generating Themes:* Codes were clustered into broader concepts (e.g., “health is inherited,” “fear of hospital,” “taboo food”).
4. *Reviewing Themes*: Codebooks were refined by cross-checking against original transcripts.
5. *Defining and Naming Themes*: Key themes were finalized, including “Cultural Heredity Logic,” “Food Restrictions,” “Knowledge Transfer Gaps,” and “Distrust of Outsiders.”
6. *Reporting*: Themes were contextualized within the Baduy cultural worldview and linked to programmatic failure in stunting eradication (Figure 5).

Throughout this process, reflexive memoing and thematic mapping tools within NVivo were used to track analytic decisions and conceptual growth. A second researcher reviewed 20% of the coded transcripts for inter-coder reliability (agreement: 92%), and discrepancies were resolved through discussion.

### 3.5 Data validation measurement

To ensure the validity and trustworthiness of the qualitative findings, several rigorous procedures were applied throughout the research process. First, inter-coder agreement was established during the thematic analysis by involving multiple researchers in the coding process; initial codes were independently generated and then compared, discussed, and refined until consensus was reached, reducing individual interpretive bias. Second, data triangulation was employed by cross-checking interview data with field observations, informal discussions with traditional leaders (*jaro*), and relevant policy and health documents, allowing themes to be validated across multiple sources. Third, researcher reflexivity was continuously practiced, particularly given the cultural sensitivity of the Baduy context. The researchers maintained reflexive field notes to critically reflect on their positionality, assumptions, and interactions with participants, including customary authorities and community members. Engagement with *jaro* and local facilitators also functioned as a form of cultural validation, ensuring that interpretations did not misrepresent customary meanings or practices. Together, these procedures strengthened the credibility, dependability, and analytical rigor of the study.

## 4 Results

### 4.1 The interview results hierarchical map

**Figure 6:**
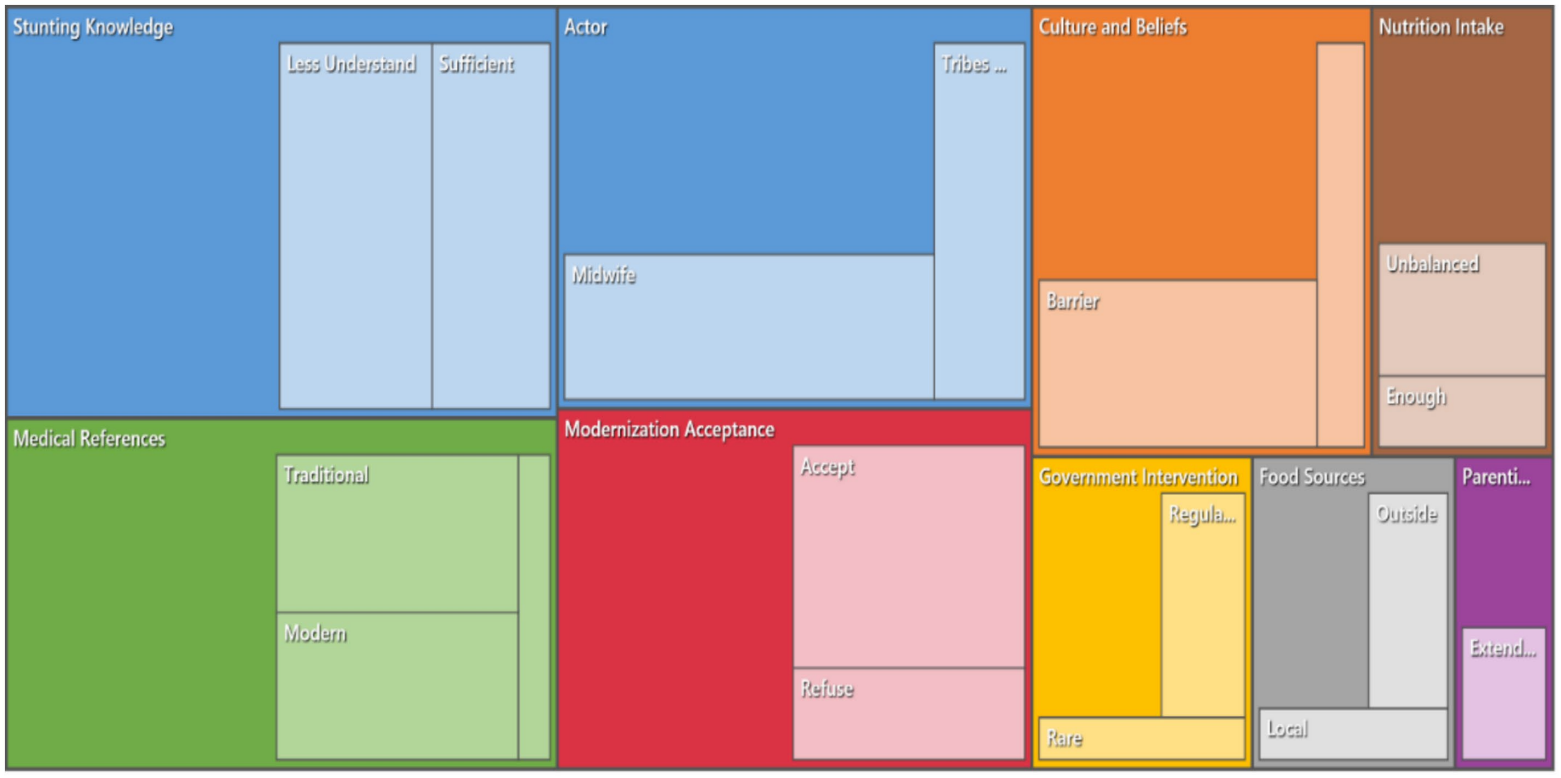
NVivo hierarchical maps based on the interview results.

The first output from the NVivo analysis is the hierarchical map *(treemap)* based on the nodes. From the analysis, identified primary themes, and subthemes, includes: (1) *Stunting Knowledge*, which gathered the local perceptions of short stature; (2) *Medical References*, reflecting the use of traditional treatment method versus modern health services; (3) *Modernization Acceptance*, indicating varying degrees of receptivity to state-led health programs; (4) *Cultural Beliefs*, which include inherited spiritual and customary explanations for child health and illness; (5) *Nutritional Intake*, addressing both the quantity and quality of food consumed by children; (6) *Parenting Patterns*, focusing on maternal knowledge, feeding practices, and gendered decision-making; (7) *Government Intervention*, related to the presence, effectiveness, and perceived legitimacy of state and NGO programs; (8) *Food Sources*, showing reliance on subsistence agriculture and cultural restrictions on market-based foods; and (9) *Health Actor Roles*, highlighting the influence of midwives, shaman (traditional birth attendants), village leaders, and husbands in health-related decisions (Figure 6).

### 4.2 The interview results cluster analysis

**Figure 7:**
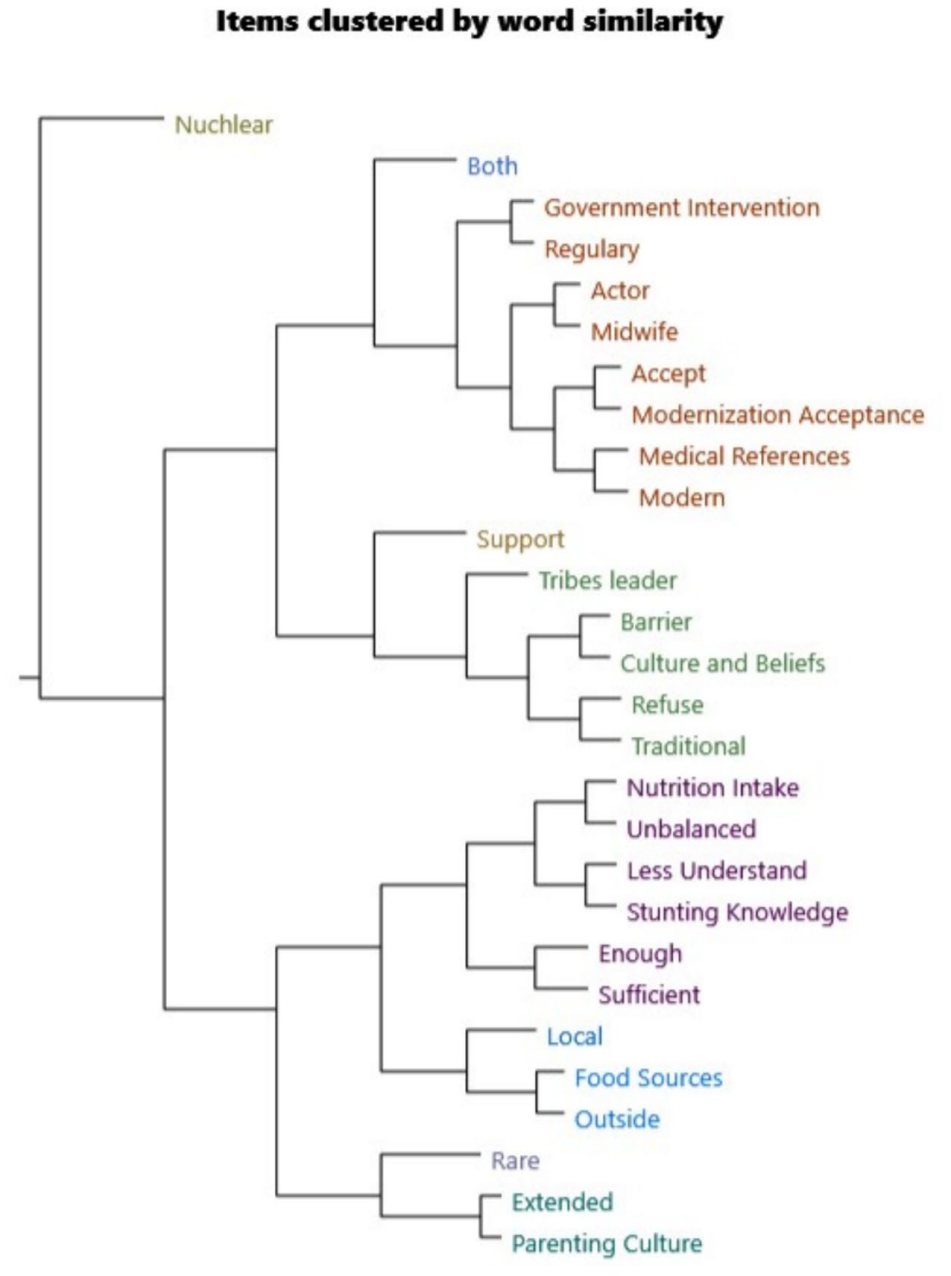
Item clustered item analyzed by using the NVivo 12 Pro.

The Pearson correlation coefficients were used to eliminate word similarity among nodes, which showed a statistically significant relationship between governmental and health governance aspects, specifically the relationship between *“Government Intervention,” “Regulatory,” “Actor,” “Midwife,”* and *“Accept.”* The high correlation coefficients (r > 0.75), suggest that the active engagement of external health actors—such as government officials and midwives—correlates strongly with community receptivity to the health interventions. These findings corroborate extant literature, notably the work of Keelan et al. (2021) and Jongen et al. (2023), which found that culturally sensitive, participatory approaches—particularly those involving trusted community figures—are important to improve the acceptance of the health intervention efforts in indigenous populations (Jongen et al., 2023; Keelan et al., 2021). Furthermore, the word cluster, which includes: *“Support,” “Tribes Leader,”* “Culture and Beliefs,” and *“Refuse,”* indicates that the traditional authority figures and cultural practices are significantly linked in shaping health-related behaviours. These findings align with the previous research conducted by Durrance-Bagale et al. (2022), which emphasizes that tribal elders and community leaders serve as pivotal gatekeepers in determining the community’s receptiveness to health initiatives (Durrance-Bagale et al., 2022) (Figure 7).

Secondly, the relation between the nutritional status and knowledge, which is reflected by the words, such as: *“Nutrition Intake,” “Unbalanced,” “Less Understand,” and “Stunting Knowledge”* also showed a moderate to strong positive correlation. The Empirical evidence from Koh et al. (2025) demonstrates that targeted nutritional education tailored to local cultural contexts can significantly improve dietary behaviors and reduce malnutrition rates (Koh et al., 2025). Later, the significant relation between the word *“Support” and “Local Food Sources”* further emphasizes that local food systems, indigenous dietary customs, and external influences are interconnected, affecting consumption patterns.

Lastly, the identified relation between the words: *“Rare,” “Extended,”* and “*Parenting Culture,”* which present correlation coefficients in the range of 0.5 to 0.65, indicating moderate relationships. These themes highlight that the extended familial support is less significant but has a moderate influence on health behaviors and nutritional outcomes. This result is supported by the previous research conducted by Michaelson et al. (2021), who advocate for the integration of extended family networks into health promotion strategies, increasing the culturally acceptable and sustainable behavior change (Michaelson et al., 2021).

### 4.3 Wordcloud analysis

**Figure 8:**
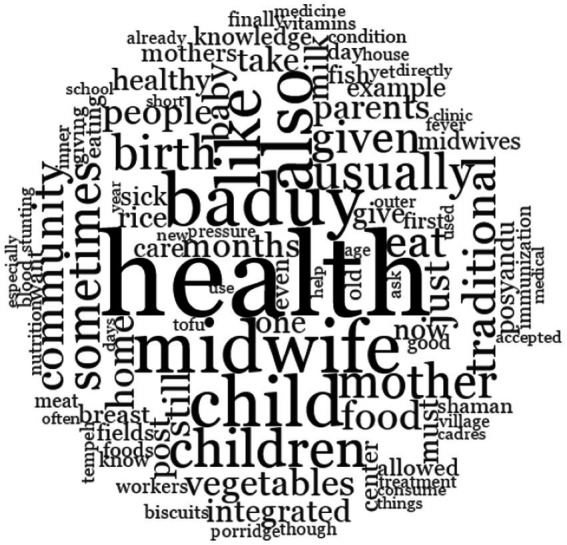
Wordcloud analysis from the nodes, exported from NVivo software.

Based on the word cloud analysis, the highest coverage percentage words are: “health,” “child,” and “Baduy,” which together align with discussions on community health priorities and child well-being, representing approximately 18.4% coverage. The prominence of “stunting” in conjunction with “health” reflects the thematic emphasis on stunting knowledge and its health implications, contributing to 18.6% coverage. The frequent appearance of “midwife” indicates the centrality of health actors in community interventions, corresponding to 17.4% coverage. Feeding-related terminology—such as “food,” “vegetables,” “milk,” “breast,” “rice,” and “meat”—matches discourse on nutritional sources and dietary practices, accounting for 17.9% coverage, while references to “parents,” “mother,” and “children” link directly to parenting patterns and caregiving roles, reflected at 17.3% coverage. The co-occurrence of “traditional” with biomedical terms like “clinic,” “medicine,” and “treatment” illustrates the integration of indigenous belief systems with modern healthcare, marking 16.7% coverage for cultural beliefs and 16.2% for medical references. Government and institutional involvement are indirectly signaled through mentions of “integrated” and “community,” achieving a peak thematic coverage of 19.3%, underscoring the prominence of healthcare system discourse. Finally, the visible balance between traditional healing practices and modern medical acceptance suggests 17.3% coverage for themes of modernization acceptance, revealing the coexistence—and occasional tension—between cultural norms and biomedical health approaches in the Baduy context (Figure 8).

### 4.4 Interview data analysis

Interview findings highlight several interconnected patterns. *First,* although formal education is culturally discouraged, knowledge about stunting (beke) is shared through informal family networks, traditional authorities, and limited external interactions. *Second*, health-seeking behavior follows a hierarchical pattern, beginning with home care and traditional remedies, then consulting shamans, and eventually accessing midwives or health centers when conditions do not improve. *Third,* acceptance of modern health services is shown through regular participation in *Posyandu* (public clinic) activities, especially in Baduy Luar communities, while the use of modern technology remains geographically and culturally selective. *Fourth,* cultural beliefs and rituals related to childbirth and childcare coexist with biomedical practices but may delay prompt medical intervention in emergencies. *Finally,* nutritional practices are influenced by subsistence farming, cultural food restrictions, and limited meals, and government health programs work best when closely collaborating with traditional leaders *(jaro)*.

#### 4.4.1 Stunting knowledge of the Baduy indigenous community

The community’s understanding of stunting has evolved significantly through health education interventions. Locally, stunting is commonly referred to as *“beke”* (short stature). Furthermore, based on the analysis of the interview results with the highest coverage level, the first and most known cultural barrier is the prohibition of formal education, which shapes knowledge transmission patterns, as explained by an informant, as follows:

*“There is no formal education available in the area, and no schools have been established. Those who can read and write have generally learned these skills from their parents, or, more frequently, from tourists visiting the villages.”–* DD, 21

The statement was also supported by a staff member who works in the district offices, as he stated as follows:

*“The Baduy people do not receive formal schooling, as their cultural norms prohibit it. The Jaro (tribal leader) explains that schools are believed to threaten and potentially erode their cultural values.”*—PUS, 33

The prohibition of formal education also shapes the Baduy community’s perceptions. Among most members, stunting is attributed to a traditional concept referred to as *beke*, which is often linked to parental occupational factors. As explained by a local shaman:

*“Children are short because parents often carry heavy loads or work hard, affecting the child's growth downward rather than upward.”* - SHA, 53

The statement was also supported by a mother with 2 children, who stated:

*“It could be hereditary factors from their parents or health factors too.”*—SK, 29

From the interview results above, the stunting knowledge domain reveals a complex epistemological transformation occurring within educational constraints. Although formal schooling is prohibited as part of cultural preservation, the community established an alternative mode of knowledge acquisition that enables the selective integration of biomedical concepts within a traditional epistemological framework. This represents what Bourdieu would term *“cultural capital”* transformation, where new forms of health literacy emerge through informal networks and social interactions rather than institutional education (Bourdieu, 2011).

#### 4.4.2 Baduy tribes medical references

Based on the word coverage analysis conducted by NVivo software, the medical references which trusted by the Baduy indigenous community are quite varied, which can be described as follows:

*“Since midwives arrived, my main function (as shaman) is more toward protecting from unwanted things, because midwives cannot do this.”*—SHA, 53

Another informant, also explaining the collaborative relationship between the shaman and midwife, also happens in their everyday lives, with his statement as follows:

*“Usually if my children were sick, I will ask to the parents first, because it's the first time for me to having and caring for a child... then if the needed, I will go to the closest family who can help us, then to the shaman, sometimes the shaman and midwife are waiting in the head of tribes houses.”*—JM, 23

The statement is supported by another informant, who is a father with 2 children, who confirmed this approach:

“*When my child has fever or cough, I make tuak (traditional drink from the local leaves) from the forest, usually for cough I give talas tuak, and if the condition not getting better, I will ask help from shaman together with midwife will bring my children to the nearest clinic or to Dr. Kasmojo* (the nearest doctor who stay in Baduy area).”—AH, 26

Healthcare utilization follows a stepped approach, as described by a father with one child, who stated:

*"First treat ourselves, ask parents first, then wait up to a maximum of 3 days, if there's no change, then take to the health center.”-* JM, 23

The statement is also supported by a mother, who stated:

*"After that, we wait for development for one to two days, if not healed, then we take them to the health centre with the help of the shaman.”–* NIS, 20

The community exhibits a hierarchical approach to health-seeking behavior, prioritizing traditional remedies before accessing modern healthcare. This pattern reflects cultural continuity rather than rejection of biomedical services. Traditional medicinal plants remain central to primary healthcare, including kunyit (*Curcuma longa*), jahe (*Zingiber officinale*), kencur (*Kaempferia galanga*), and various leaves like kacapiring (*Gardenia jasminoides*) and sirih (*Piper betle*).

#### 4.4.3 Baduy tribes health modernization acceptance

The spatial centrality and font size of these keywords in the visualization affirm the quantitative NVivo coverage, demonstrating a coherent match between thematic prominence and visual representation. The high percentage coverage can be described as follows:

*“Yes, I often attend, never miss from the first child until now, usually once a month, organized by the Posyandu.*”—SK, 24

Health authorities also noted this engagement trend. As the local health centre director stated:

*“Now, when there are Posyandu activities, they come by themselves to the Posyandu posts.”*—VS, 63

This observation is supported by a midwife, who highlighted a shift in health awareness:

“*Almost all residents here have started to open their minds and begun to understand health.”*—NI, 32

Technological adoption, however, varies greatly depending on individual preferences and living location. A tour guide explained how some community members actively access mobile communication:

*“They will go down to Ciboleger Terminal (local bus terminal), or to other nearby villages... They will pay two thousand rupiah to charge their mobile phones, without time limits.”*—AL, 24

Those living deeper in the forest—particularly in Baduy Dalam—tend to be more resistant to technological change, maintaining stronger adherence to traditional restrictions. As the same informant emphasized:

*“Absolutely not allowed to photograph in Baduy Dalam. But if photographing Baduy Dalam residents when they are not in Baduy Dalam, that is allowed.*”—AL, 24

These accounts illustrate that participation in Posyandu activities and the adoption of technology are negotiated processes, shaped by personal choice and spatial location. While Baduy Luar residents may integrate biomedical engagement and limited technological use into daily life, deeper forest communities maintain stricter cultural boundaries. This pattern demonstrates a localized balance between modernization and cultural preservation, where integration is selective and deeply rooted in place-based values.

#### 4.4.4 Baduy tribes cultural beliefs on health intervention

Spiritual and customary explanations for child health remain integral to the community’s worldview. Birth rituals include seven-day naming ceremonies, protective cord placement around infants’ waists, and specific postpartum confinement practices. These beliefs coexist with biomedical understanding rather than competing with it. Traditional birth attendants described spiritual roles:

*“Every paraji (traditional shaman) here has their own 'spiritual holding'... sometimes there are supernatural disturbances like old women, figures who have passed away, which are called kuntilanak.”*—AN, 32

A young community member supported and explained protective rituals:

*“There's a celebration and prayers, then the baby is given a belt made of black thread, used to avoid disease.”*—MER, 13

A Young man confirmed the baby naming ceremonies, which have already been performed for hundreds of years in Baduy, as stated:

*“There's a small celebration usually with giving a waist necklace from thread that has been prayed over by the elder.”*—JAR, 23

A shaman also confirmed the previously given information, which is:

“*As far as I know, customs regarding mothers and children include circumcision, then the custom that 7 days after giving birth, you can't leave the house.”—*SHA, 53

A community member from Baduy Dalam explained spiritual consultations:

*“When someone is sick, using traditional medicine to treat and asking for spiritual guidance. Asking for guidance from ancestors through intermediaries of respected elders.”*—OD, 35

However, the customary requirement that mothers remain indoors for 7 days postpartum—while culturally meaningful as a period of rest and spiritual safeguarding—can inadvertently hinder timely access to biomedical care. In cases of postpartum complications such as haemorrhage, infection, or neonatal distress, the delay in seeking formal medical assistance significantly heightens health risks for both mother and child. This spatial and temporal restriction, when combined with the community’s limited integration of external healthcare providers, poses a critical challenge for maternal–child health interventions in the Baduy context.

#### 4.4.5 Baduy tribes nutritional intake

Dietary patterns reveal both cultural preferences and economic constraints affecting child nutrition. While traditional diets consist primarily of rice, vegetables, fish, tempeh, and tubers, feeding frequency frequently remains below recommended standards, as children typically eat only one to two times per day instead of the advised three meals.

A Mother with a daughter reported concerning feeding patterns:

*“My child has difficulty eating, not regularly like other children, at most once a day, and that's after making fried rice first because white rice is rarely wanted.”*—AL, 24

The statement was supported by another mother, who stated her daughter has an irregular eating habit, which she stated:

“*My daughter usually eats 2-3 times a day, sometimes only 2 times a day because given boiled tubers or corn, so they're full.”*—DD, 21

Furthermore, a young community member, who is only 13 years old, confirmed that her eating habits are also irregular, because of the limited meal frequency:

*"For eating twice, a day, morning and afternoon, during the day, just eating snacks like cassava if available.”*—MER, 13

Complementary feeding practices show traditional approaches. A young father noted that a newborn baby is introduced to the meals early, as stated:

*"If my baby is already 7 days old and wants a banana, we give a banana and chewed rice as desired. For vegetables, when a bit older, usually 3 months.”*—JM, 23

Another mother also supports that statement, that:

*“At 7 or 8 months, given food usually alternating with breastmilk... first is banana, biscuits, but that's just snacks.”*—DD, 21

The nutritional intake domain reveals critical gaps between recommended feeding practices and actual behaviours that directly contribute to stunting risk. The predominance of 1–2 daily meals instead of the recommended 3 + meals represents a significant risk factor for growth faltering, particularly when combined with high physical activity levels in agricultural work. The early introduction of solid foods at 7 days of age contradicts WHO guidelines for exclusive breastfeeding until 6 months, potentially increasing infection risk and reducing breast milk intake. However, the use of pre-masticated foods may enhance digestibility and nutrient absorption, representing traditional knowledge that requires careful evaluation rather than wholesale rejection.

The reliance on energy-dense but nutrient-poor foods (rice, tubers) reflects both cultural preferences and economic constraints. The preference for processed/fried preparations over plain rice suggests children’s natural preference for more palatable foods, indicating opportunities for improving nutrition through food preparation modifications rather than complete dietary changes.

#### 4.4.6 Parenting patterns

Maternal knowledge and feeding practices demonstrate gendered decision-making patterns within traditional family structures. Mothers primarily manage daily childcare and feeding decisions, while fathers control resource allocation and healthcare access. Extended family involvement, particularly grandmothers, provides crucial support and knowledge transmission.

A Mother described family support systems:

*"There's husband when at home or to grandfather and grandmother because they're close, behind the house, neighbours usually.”*—AL, 24

A young father also explained collaborative care:

*"Often taking turns, sometimes with father, sometimes with mother, sometimes when both are busy, the in-laws also help watch.”*—AS, 23

Breastfeeding duration typically extends 1–2 years. As explained by one of the mother, who reported:

*“Giving breastmilk from birth until 1 year old for the first child, the second one is still breastfeeding because still 9 months old.”—*AL, 24

Decision-making authority varies by gender and generation. A mother, described consultation patterns:

*“Asking mother, that's when first having a child, asking mother a lot... because I don't understand yet about caring for children.”*—OD, 21

The pattern also confirmed by a father, who said about the elder authority in Baduy community:

*“Often, always ask parents first.”*—AS, 23

The parenting patterns domain reveals complex intergenerational knowledge transmission systems that both support and potentially constrain optimal child care practices. The strong extended family involvement provides crucial social capital that buffers against parenting stress and knowledge gaps, particularly important given the young maternal age (average 20 years) and educational restrictions. Hierarchical patterns of decision-making, although socially supportive, can reinforce suboptimal health practices when the authority of elders supersedes evidence-based guidance. The predominance of maternal responsibility for daily care, combined with patriarchal authority over resource allocation, creates potential barriers to accessing healthcare services and implementing nutrition interventions. Breastfeeding duration patterns align well with WHO recommendations (2 + years), representing a positive traditional practice that supports child nutrition and development. However, the integration of complementary feeding within extended family decision-making may complicate nutrition education efforts, requiring interventions that engage multiple generations rather than focusing solely on mothers.

#### 4.4.7 Government intervention

The spatial centrality and font size of these keywords in the visualization affirm the quantitative NVivo coverage, demonstrating a coherent match between thematic prominence and visual representation. State health programs demonstrate significant penetration and acceptance within Baduy Luar communities. The Posyandu system operates effectively, providing monthly health monitoring, immunization, and nutrition supplementation. Community response has evolved from initial resistance to active participation.

A local health center director described program implementation:

*"Health centers conduct socialization or counselling programs related to stunting through village midwives integrated in Posyandu posts.”*—VS, 63

A local Midwife, also explained about the service delivery:

*"Weighing, immunization, Supplementary Feeding Program (PMT), sometimes there are also mothers who want free blood pressure checks.”*—FP, 32

A mother also described the services received:

*"Mostly measuring, weighing, checking the child and mother's health, giving immune injections, then giving guidance.”*—AL, 24

Collaborative governance emerges through partnerships with traditional leaders. The health center director emphasized:

*“At minimum, if we want to conduct any activities related to the Baduy community, we must inform at least the village head or jaro first.”*—FP, 32

A traditional healer confirmed this requirement:

*"All activities related to the Baduy community... the customary rule is to ask permission first... permission to the village head or commonly called jaro.”*—ER, 53

The government intervention domain demonstrates successful integration of state health programs within indigenous governance structures through collaborative rather than imposed implementation approaches. The requirement for traditional leader approval before program implementation represents recognition of indigenous sovereignty that enhances rather than undermines program effectiveness. The differential success rates between basic services (weighing, immunization) and complex interventions (PMT feeding programs) reflect both geographic constraints and programmatic design limitations. The high acceptance of Posyandu services suggests that when interventions are culturally appropriate and demonstrate clear benefits, indigenous communities can achieve coverage rates exceeding national averages. The resource constraints limiting program reach highlight the need for adaptive implementation strategies that account for subsistence agricultural lifestyles and geographic isolation. The collaborative governance model provides a template for indigenous health program implementation that respects cultural autonomy while achieving public health objectives.

#### 4.4.8 Food sources

Food security relies primarily on subsistence agriculture, with rice cultivation forming the economic and nutritional foundation. Households maintain traditional food production through huma (dry rice fields) and forest gathering, supplemented by market purchases for protein sources and processed foods.

Multiple informants described mixed food sourcing patterns, confirmed by the other informant, who explained:

*"We are relying on our own gardens, but for vegetables like soup, tempeh, tofu, chicken, fish, and onions, we purchase from the local traders. However, food like meat and eggs is expensive and prohibited in our area, and there is no equipment to keep our food fresh, so we are worried about our food spoiling quickly.”*– AH, 26

Cultural food restrictions shape dietary diversity. A community member from Baduy dalam, explained:

*"Rice is the staple food, while durian is the main fruit consumed, though only occasionally, due to cultural prohibitions. The rearing or consumption of four-legged animals, such as goats and cows, is prohibited in the Baduy region under the directives of the tribal leader.”*—GIU, 28

A young man, confirmed restrictions:

*“We obtain our food from the fields and usually eat only once a day, sometimes with steamed sugarcane. Occasionally, my parents buy vegetables from itinerant sellers. We seldom consume meat; our main source of animal protein is salted fish, as I go to the market only once a week, and salted fish is the only type that can be preserved for that duration.”*—JM, 23

Seasonal food availability impacts nutritional quality; as established, most Baduy people eat salted fish for their daily meals, as mentioned by a mother with two daughters, as follows:

*"Most of our food is purchased from the market, although we occasionally obtain vegetables from our garden. Items such as tempeh, tofu, and salted fish are typically bought at the market. My daughter and I frequently eat salted fish with rice, and my husband particularly enjoys it whenever he returns from the fields.”*– ER, 53

The food sources domain reveals a complex food system that combines subsistence agriculture with market integration, creating both opportunities and constraints for optimal child nutrition. The heavy reliance on rice as a staple crop provides energy security but may limit dietary diversity necessary for preventing micronutrient deficiencies associated with stunting. The cultural restrictions on livestock raising while permitting consumption create economic barriers to accessing animal protein, potentially contributing to protein-energy malnutrition. However, the integration of plant-based proteins (tempeh, tofu) demonstrates adaptive strategies that can meet nutritional needs within cultural constraints.

#### 4.4.9 Health actor roles

The spatial centrality and font size of these keywords in the visualization affirm the quantitative NVivo coverage, demonstrating a coherent match between thematic prominence and visual representation.

Multiple actors influence health-related decisions within complex social networks. Midwives serve as primary biomedical authorities, providing clinical services while navigating cultural sensitivities. Their role extends beyond clinical care to health education and community mobilization.

A local Midwife, described her comprehensive role:

*"From us equipping cadres like weighing, measuring height... for handling minor illnesses, we just give guidance for first aid.”*—FP, 32

A local health center director, also explained about the collaborative approach:

*"Village midwives will approach the community first... village midwives will make special approaches to Baduy communities.”*—VS, 63

Traditional healer, also noted the evolution of relationships:

*"Thank God until now when village midwives come to their location, they come by themselves without having to be asked.”*—SHA, 53

Village leaders *(jaro)* function as cultural gatekeepers. The health center director emphasized their authority:

*“Jaro or traditional leaders are highly followed by the community, so if there are any programs... at minimum must get permission from these traditional leaders.”*—VS, 63

A traditional healer confirmed this process:

*“Later, if we already have permission from Jaro or the village head, then we can coordinate with parties to be involved in activities.”*—JRO, 45

Staff member described the governance structure:

*“Customary institutions that handle implementation activities. From head to Jaro. Handling all kinds of customary law.”*—PUS, 33

A father from the baduy community also explained his role as follows:

*"The wife knows more, but if there's advice or health guidance from midwives, there definitely is.”*—DD, 21

The health actor roles domain reveals a complex network of formal and informal health authorities that operate through negotiated relationships rather than hierarchical command structures. The successful integration of midwives with traditional birth attendants demonstrates how professional complementarity can enhance rather than replace indigenous health systems. The gatekeeping function exercised by traditional leaders (*jaro*) represents both a constraint and an opportunity for health program implementation. While requiring additional negotiation steps, this system ensures cultural appropriateness and community buy-in that enhances program sustainability. Evidence of high coverage under collaborative approaches further indicates that engagement within Indigenous governance structures is more effective than efforts to circumvent them.

### 4.5 Identified relation between interview nodes

**Figure 9:**
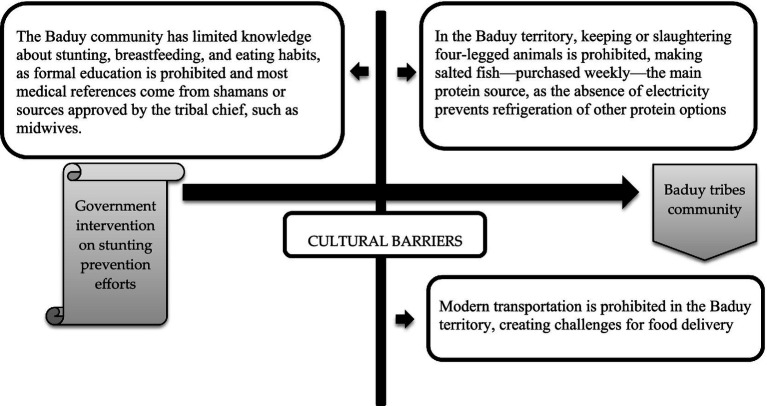
Relationship between identified factors in stunting prevention nodes.

The network diagram illustrates a complex web of interrelated themes centered around the key concepts of “Actor” and “Culture and Beliefs,” highlighting the critical role of community actors and cultural norms in shaping health-related behaviors and attitudes. The high degree of interconnection, evidenced by the clustering of elements such as “Government Intervention,” “Food Sources,” “Nutrition Intake,” and “Stunting Knowledge,” underscores the multifaceted nature of health interventions within traditional communities (Figure 9). The strong relationships among these themes suggest that community actors, including traditional leaders and health personnel, serve as vital mediators in the dissemination of health information, the acceptance of modern health practices, and the promotion of nutritional behaviors, aligning with findings by Rahmawati et al., (2021). Moreover, the prominent role of “Culture and Beliefs” suggests that a deeply ingrained cultural identity has a significant influence on perceptions of health and nutrition, necessitating culturally sensitive strategies for effective intervention. The interdependencies depicted in the diagram underscore the need for health promotion programs to address the social authority of local actors, alongside the cultural context, in order to facilitate behavior change and improve health outcomes, particularly within Indigenous communities where tradition and hierarchy significantly influence decision-making.

## 5 Discussion

Our findings from the interviews suggest the need for an analytical lens that can account for the complex relationships between historical trajectories, cultural norms, and structural constraints to explain the persistence of stunting within the Baduy communities. The theoretical framework of Pierre Bourdieu provides a compelling viewpoint for analysing these dynamics, especially his ideas of habitus, field, capital, doxa, and symbolic violence (Bourdieu, 1989). First, from the interview results, centuries of ecological adaptation, lived experience, and rigorous adherence to *adat* (customary law) have all contributed to the Baduy habitus. The customs that have been practiced for hundreds of years show up as *embodied* behaviors, moral standards, and eating patterns that are ingrained in the community, and people hardly ever question them. According to this habit, health is not determined by biomedical measures such as the WHO growth standards or height for age. Rather, based on the interview results, it is gauged by locally significant indicators, like a child’s capacity to work in the fields, their stamina for long walks, or their general harmony with communal life. This is the practical logic of daily life, according to Bourdieu (1989): even when actions deviate from accepted scientific norms, they still make sense in their own context (Schirato and Roberts, 2020). The results were also supported by a previous research conducted by Lohmann et al. (2024) on Indigenous Arctic communities, where health evaluations are based on cultural frames rather than clinical indicators (Lohmann et al., 2024). Because their very definitions of “success” function outside the Baduy doxa, this viewpoint helps explain why external stunting prevention campaigns may be met with skepticism or indifference.

One of the clearest examples of the Baduy doxa is the prohibition against keeping four-legged livestock. While outsiders may interpret this as an agricultural policy designed to prevent crop destruction, for the Baduy tribe community, it is also a deeply symbolic act that marks their distinct cultural identity and moral boundaries. The individual’s compliance with this prohibition is a form of *symbolic capital*, which, in a short explanation, is a way of accumulating honor, respect, and legitimacy within the community (Edgerton and Roberts, 2014). Based on interview results, the *jaro* (head of tribes), as custodians of *adat,* enforce such rules not only to regulate economic life but also to preserve the cultural fabric that differentiates the Baduy from surrounding societies. However, as Bourdieu’s theory makes clear, symbolic capital in one specific field can lead to deficits in another (Swartz, 1997). In this case, the prohibition potentially removes a major source of animal protein from the local diet, which has measurable consequences for child nutrition and growth. Even though poultry and fish are permitted, the ban on slaughtering them within Baduy territory limits fresh protein availability, forcing reliance on preserved foods such as salted fish, because the distance from the Baduy territory to the nearest market is quite far, almost 7 h of walking. Daily consumption of salted protein creates a nutritional profile that is high in sodium and low in essential amino acids, contributing to chronic undernutrition (Ahmed et al., 2022). Similar patterns are observed in Titaley et al. (2021) study conducted in Maluku, Indonesia, which found that culturally rooted food restrictions undermined protein intake despite adequate caloric availability (Hedo and Katmini, 2022). Here, Bourdieu’s insight into the non-convertibility of capital is evident: symbolic capital that strengthens cultural cohesion cannot easily be translated into health capital without cultural negotiation (Bourdieu, 1989).

Furthermore, the stunting prevention effort was performed in a contested terrain where different forms of capital are “fought” for dominance. On one side are the modern figures, represented by *bidan* (midwives) and other health professionals, such as doctors and *mantri* (nurses), whose authority rests on institutionalized health capital—credentials, biomedical knowledge, and access to formal health infrastructure. On the other side is the cultural figure, represented by the *jaro* (chief of tribes) and other traditional authorities, whose power derives from symbolic and social capital accumulated over generations. While *bidan* appear more frequently in NVivo-coded interviews when discussing practical health interventions, their influence is limited by the jaro’s power to define what is culturally permissible. This is a classic case of what Bourdieu (1989) calls field autonomy: rules and capital forms from one field do not automatically hold value in another (Swartz, 1997). Without the *jaro* (chief of tribes) endorsement, modern health recommendations, such as encouraging more frequent egg consumption or early antenatal visits, may be ignored or subtly resisted. This pattern is also supported in the research conducted by Kumalasari et al. (2020) on West Papuan communities (Tehit and Yaben tribes community), where health advice only gained traction when reframed to resonate with local cosmologies (Kumalasari et al., 2020). Thus, the challenge is not only disseminating *“correct”* health information but translating it into the symbolic language of the cultural field.

Mobility restrictions offer another perspective. Whereas the prohibition against motorized transport is not a random inconvenience but a moral boundary embedded in the Baduy habits. Walking long distances to trade, procure goods, or visit health services is a valued practice that affirms cultural integrity and environmental stewardship. From the perspective of the cultural field, rejecting motorized transport is a form of symbolic capital accumulation—it signals loyalty to *adat* and rejection of external dependency. Yet, from the perspective of the healthcare field, these mobility barriers significantly reduce access to fresh, perishable protein and healthcare services, especially for pregnant women and young children. Bourdieu’s notion of misrecognition is relevant here: the symbolic value attached to physical hardship obscures the material consequences for nutrition and health (Schirato and Roberts, 2020). The findings are also relevant to the study conducted by Ferguson et al. (2017), on study at the remote Aboriginal communities territory in Australia, which documented similar dynamics where cultural restrictions on transport reinforced dietary dependence on shelf-stable, low-nutrient foods, with predictable consequences for health outcomes (Ferguson et al., 2017).

Later on, the marriage practices within the Baduy further demonstrate how the intergenerational reproduction of habitus can perpetuate structural health vulnerabilities. The arrangement of marriages before birth and the tendency for girls to marry soon after menarche are deeply embedded kinship strategies aimed at consolidating social capital and maintaining cultural purity. From a biomedical standpoint, adolescent pregnancy is a well-established risk factor for low birth weight, maternal depletion, and intergenerational stunting (Nafisah and Astuti, 2023). Yet within the Baduy cultural field, these practices are seen as honorable and dutiful, aligning with the symbolic capital of fulfilling family obligations. This is where symbolic violence, another of Bourdieu’s key concepts, comes into play: young women internalize and accept norms that limit their autonomy, educational attainment, and nutritional status, believing them to be natural and desirable (Harrison et al., 2024). The subtlety of this form of domination is that it operates without overt coercion—individuals willingly enact practices that ultimately reproduce their own disadvantage.

The Baduy community’s perception of stunting as a natural or hereditary condition—often described locally as *beke* (innate shortness) or attributed to parents’ hard labor—offers a compelling illustration of Pierre Bourdieu’s concept of habitus. Within the field of Baduy customary life, the habitus is structured by a worldview that normalizes bodily outcomes as part of a divine or ancestral order, rather than as pathologies to be medically corrected. This interpretative framework functions as a form of cultural capital that maintains social cohesion and spiritual harmony, allowing conditions like stunting to be assimilated into everyday experience without stigma or crisis. When biomedical actors introduce the concept of stunting as a preventable nutritional deficit, they are effectively operating within a different field—one governed by scientific rationality and public health metrics—where a contrasting habitus prioritizes intervention and optimization. The resulting disconnect is not merely a “knowledge gap” but a clash of symbolic universes, wherein the same physical reality is accorded divergent meanings and values depending on the field in which it is understood.

Furthermore, the Baduy community’s adherence to food taboos, preference for traditional medicine, and reliance on collective deliberation underscore how cultural capital is unevenly distributed and gendered across social spaces. Food restrictions during *Kawalu* or prohibitions on certain meats are not arbitrary rules but symbolic capital that reinforces identity and spiritual integrity, often at the expense of biomedical nutrition guidelines. Similarly, the choice to consult shamans and use herbal remedies before seeking clinical care reflects an embodied cultural capital passed down through generations, which holds more immediate legitimacy within the domestic and traditional fields. Importantly, health-related practices are deeply segmented by gender: women accumulate capital in reproductive care, child-feeding, and herbal knowledge, while men hold capital in communal leadership, customary law, and economic provision. This gendered division of habitus means that interventions targeting only mothers—such as Posyandu sessions—may improve individual biomedical capital but fail to shift household-level decisions, which are often mediated by male authority and collective deliberation. Effective health promotion therefore requires engaging with the field’s power structures—including traditional leaders like the *Jaro*—and facilitating the conversion of traditional capital into forms that align with health objectives, rather than bypassing or devaluing the deep-seated logics that organize Baduy social life.

**Figure 10:**
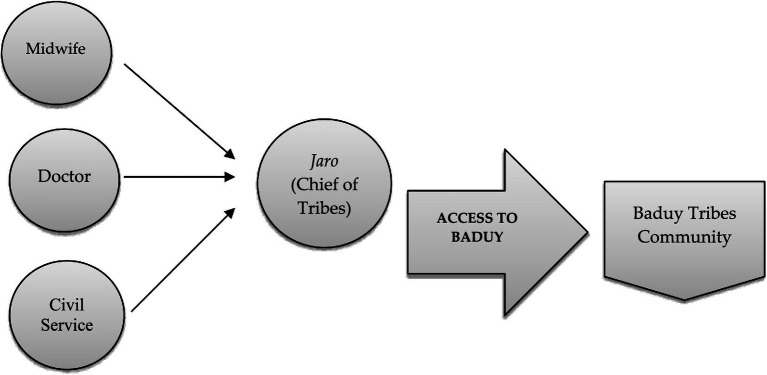
The schemes of proposed stakeholder cooperation in implementing the stunting prevention policy.

Furthermore, based on the research conducted by Winarti et al. (2024), recommended that one of the proper solutions for the stunting prevalence, especially for the remote tribes in Indonesia, is the special biscuit, enriched with protein and a long expiration date, which is produced by the government (Winarti et al., 2024). However, the governance dimension adds an additional structural constraint. The recent revelations by the KPK (the corruption eradication commission) regarding corruption in the national PMT biscuit program (2016–2020) illustrate how political and economic capital can be misappropriated in ways that undermine public trust (Akmal, 2025). The KPK’s findings—that the nutritional quality of biscuits was deliberately reduced by replacing key nutrient premixes with cheaper sugar and flour—are not merely an administrative failure but a symbolic injury to public health credibility. In Bourdieu’s terms, this represents a loss of symbolic capital for the public health field, making it harder for state actors to exert legitimate influence in communities like the Baduy (Bourdieu, 2011). The study by Gillespie et al. (2019) on nutrition governance highlights that once trust in state-led nutrition programs is eroded, communities often retreat into self-reliance, even when it means relying on nutritionally suboptimal practices (Gillespie and Nisbett, 2019). For the Baduy, such scandals likely reinforce pre-existing skepticism toward external interventions, making the *jaro-*led cultural field even more dominant in shaping local health practices. Our research also proposed whether every policy on stunting prevention needs to be led, and collaborate with the *Jaro* to get access to the Baduy tribes’ community (Figure 10).

When these findings are also supported by other studies, such as Suryawan (2025). on the Kasepuhan in West Java (Suryawan, 2025), Kumalasari et al. (2020) on West Papuan feeding taboos, and Rohmatullayaly et al. (2025) on Baduy child growth norms—a coherent pattern emerges: stunting in indigenous contexts is rarely the result of food scarcity alone (Kumalasari et al., 2020; Rohmatullayaly et al., 2025). Instead, it reflects a durable alignment between habitus, symbolic capital, and field structures that prioritize cultural survival over biomedical optimization. These practices are not irrational; rather, they are rational within the logic of their own field. The real challenge for policy is to design interventions that respect and preserve symbolic capital while enhancing health capital. Also, in short, the perspective of the Baduy tribe community, based on specific analytical themes, can be described as follows:

Based on the research data, beyond cultural norms, the distribution of necessities in the Baduy community is further constrained by structural and material factors that interact with customary rules. Limited market access, due to the prohibition of modern transportation within Baduy territory, significantly restricts the frequency and volume of food supplies entering the community. Most households rely on walking long distances to reach nearby markets, which reduces purchasing power and limits access to perishable foods such as fresh meat, eggs, and vegetables. Animal-based protein is not only culturally restricted but also economically inaccessible, as prices for fish and meat obtained from outside the territory are relatively high compared to household incomes, leading families to prioritize cheaper, storable staples. Food storage presents an additional challenge: the absence of refrigeration and modern preservation technologies means that protein-rich foods cannot be stored safely for extended periods, reinforcing dependence on salted or dried fish. These constraints collectively shape everyday food choices and exacerbate nutritional vulnerability, particularly for young children and pregnant women. When viewed through Bourdieu’s concept of field, these material limitations intersect with symbolic and cultural capital, demonstrating that stunting is produced not only by beliefs and practices but also by unequal access to resources embedded within the broader socio-economic and infrastructural context (Grenfell, 2008).

The data presented in Table 3 demonstrate that food practices among the Baduy community are not merely individual dietary choices but are socially regulated expressions of habitus and cultural capital. Food taboos, particularly the prohibition on keeping and slaughtering four-legged animals, are deeply embedded in pikukuh adat and function as embodied cultural capital that signals moral integrity, obedience to ancestral rules, and collective identity. While these practices generate symbolic capital within the customary field, they simultaneously restrict access to animal-based protein and essential micronutrients, contributing to nutritionally monotonous diets dominated by cassava, rice, and forest vegetables. The avoidance of fortified and processed foods further illustrates the tension between the biomedical field and the customary field, where external nutrition interventions lack legitimacy because they are perceived as incompatible with cultural values. In parallel, the preference for traditional medicine over biomedical treatment reinforces the authority of traditional healers, who possess greater symbolic capital than health workers such as bidan, leading to delayed recognition of growth faltering. Importantly, these food-related practices and health-seeking behaviors are collectively governed through customary deliberation involving elders and jaro, which limits individual maternal agency and constrains the adoption of recommended infant and young child feeding practices. Taken together, the table illustrates how stunting among the Baduy is socially reproduced through culturally valued practices operating within a specific power structure, underscoring that effective nutrition interventions must engage with existing cultural capital rather than attempt to replace it.

**Table 3:** Key summary of findings based on specific analytical themes.

Analytical theme	Quotes
Meaning of health	*“Jalingeur (agile) looks like a child who is not agile is sick… in Baduy, they are all healthy, strong, and rarely sick. My father said, ‘Do not complain; just because you have a fever is nothing, as long as you can walk—Ulah Angluhan (do not complain).’”*
Perception of stunting	*“Short of height is seen as something natural, a variation created by the Almighty, not a disease.”*
Food taboos	*“During the month of Kawalu, the Baduy community fasts for 3 months and is not allowed to consume chicken eggs, and no tourists are allowed to visit Inner Baduy.”*
Experience at *Posyandu*	*“Of course, it’s really nice to gather with other mothers. We can share experiences with other mothers, with the cadres, and with the midwives, and we can consult directly.”*
Health rituals	*“When children fall ill, I avoid panic and first give traditional herbal remedies, asking for help from my husband or my mother.”*

The empirical data summarized in Table 4 indicate that child feeding practices in the Baduy community are shaped by culturally embedded norms rather than biomedical nutritional guidelines. Stunting is widely perceived as a natural or hereditary condition, which reduces parental concern and limits early responses to growth faltering, contributing to an estimated prevalence of around 60%—far above the national average. Children generally eat two to three times per day, following adult meal schedules, with little emphasis on age-specific nutritional needs or supplementary feeding. Daily diets are dominated by rice, cassava, and forest vegetables, resulting in low dietary diversity. Due to customary restrictions on raising and slaughtering four-legged animals, families rely primarily on salted or dried fish as their main source of animal protein, leading to low intake of fresh protein and essential micronutrients. Breastfeeding is culturally supported, yet complementary feeding often begins late and consists mainly of soft rice or mashed cassava, offering limited nutritional density during the critical 6–24 month growth period. Additionally, fortified foods and government nutrition supplements are frequently avoided because they are perceived as incompatible with traditional values. Together, these practices illustrate how culturally structured routines contribute to persistent nutritional vulnerability among Baduy children (Table 5).

**Table 4:** Analytical table for the food taboo implication in the Baduy community.

Food taboo/practice	Empirical description (interview findings)	Nutritional implication	Bourdieu’s conceptual analysis
Prohibition on keeping four-legged animals (e.g., cows, goats, buffalo)	Participants consistently stated that raising four-legged animals violates *pikukuh adat* and is believed to disrupt harmony between humans and nature. Livestock is seen as symbolically associated with the exploitation of land.	Limited availability of animal protein, iron, zinc, and vitamin B12—nutrients essential for child growth and prevention of stunting.	This taboo represents embodied cultural capital, where moral virtue and obedience to tradition carry symbolic value, even at the cost of nutritional adequacy.
Prohibition on slaughtering animals within the Baduy territory	Meat consumption is allowed only if obtained from outside the Baduy land, which is rare due to mobility and trade restrictions. Slaughtering animals locally is considered a serious customary violation.	Irregular and minimal intake of meat-based foods among pregnant women and young children.	Functions as symbolic capital, reinforcing cultural purity and collective identity while structurally constraining dietary diversity.
Reliance on plant-based staple foods (cassava, rice, forest vegetables)	Daily diets are dominated by cassava (*singkong*), rice, and boiled vegetables, with minimal variation. These foods are considered “clean” and culturally appropriate.	Diets are energy-sufficient but micronutrient-poor, increasing the risk of chronic malnutrition.	Reflects habitus, where food choices are normalized through long-standing practices rather than nutritional reasoning.
Avoidance of fortified or processed foods	Industrial foods (e.g., fortified biscuits, formula milk) are often rejected as *“unnatural”* or incompatible with ancestral rules.	Missed opportunities for micronutrient supplementation during critical growth periods.	Illustrates tension between the biomedical field and the customary field, where modern health interventions lack legitimacy.
Preference for traditional medicine (*obat kampung*) over biomedical treatment	Illness, including poor child growth, is frequently attributed to spiritual imbalance or destiny rather than nutritional deficiency. Traditional healers are consulted before health workers.	Delayed diagnosis and intervention for growth faltering and maternal undernutrition.	Traditional healers possess higher symbolic capital than midwives (*bidan*) within the local health field.
Collective decision-making through customary deliberation (*musyawarah adat*)	Decisions regarding child feeding, healthcare access, and external assistance require approval from elders and *jaro*. Individual mothers have limited autonomy.	Reduces flexibility in adopting recommended infant and young child feeding practices.	Demonstrates how power relations within the social field regulate health behavior and reproduce nutritional vulnerability.

**Table 5:** Observed empirical data based on the field data.

Aspect	Field findings	Observed pattern
Stunting prevalence	Many parents and elders perceive children’s short stature as a natural or hereditary trait rather than a nutrition-related problem, resulting in limited concern about growth faltering.	Stunting prevalence is estimated at around 60%, substantially higher than the national average (19.8% in 2024).
Meal frequency	Children typically consume two to three meals per day, following household eating schedules, with little distinction between adult and child nutritional needs.	Regular meals but limited frequency and lack of supplementary feeding for young children.
Staple foods	Daily meals consist mainly of rice, cassava, and boiled forest vegetables, with minimal variation across days.	Diets are energy-based but nutritionally monotonous.
Protein sources	Due to restrictions on raising and slaughtering four-legged animals, families rely mostly on salted or dried fish obtained from outside the community.	Low intake of fresh animal protein and micronutrients.
Infant feeding	Breastfeeding is culturally encouraged, but complementary feeding often begins late and consists mainly of soft rice or mashed cassava.	Limited use of protein-rich complementary foods during the critical 6–24 month period.
Fortified foods	Government-provided fortified foods and supplements are often rejected as “unnatural” or incompatible with customary rules.	Very low participation in formal nutrition programs.

From a Bourdieusian standpoint, the way forward lies in capital conversion, recasting health-promoting behaviors as sources of symbolic capital within the cultural field. This could involve reframing certain protein-rich foods, such as eggs or freshwater fish, as consistent with adat values, or introducing culturally sanctioned mobility solutions that expand market access without violating prohibitions on motorized transport. Strengthening alliances between modern health practitioners and traditional tribe leaders can create a hybrid authority structure in which biomedical and symbolic capitals are co-produced, reducing the perception of outside imposition. Without such alignment between the logics of the biomedical and cultural fields, attempts to address stunting risk are perceived as cultural threats rather than health solutions, thereby perpetuating the cycle of undernutrition.

Furthermore, the findings indicate that the processes of embodiment, objectivization, and institutionalization of cultural capital among the Baduy community have been highly effective in preserving social cohesion and traditional belief systems; however, these same processes simultaneously create structural and symbolic boundaries that limit engagement with formal health services and biomedical understandings of child growth, thereby posing significant challenges to stunting prevention efforts. This condition underscores the need for policy approaches that move beyond technocratic health interventions toward culturally embedded governance models. In this regard, Indonesia’s legal and policy framework provides a clear mandate for culturally sensitive, collaborative action. Presidential regulation no. 72 of 2021 on the Acceleration of Stunting Reduction explicitly emphasizes cross-sectoral convergence, community participation, and behavior change communication as core pillars of national stunting policy, thereby legitimizing the formal involvement of indigenous governance actors, such as jaro, in the planning and implementation of nutrition and maternal–child health programs.

Legally, the constitutional guarantee of children’s rights under Article 28B(2) of the 1945 Constitution, reinforced by Law no. 35 of 2014 on Child Protection, obliges the state to ensure optimal growth and development for all children, including those in indigenous communities, without negating their cultural autonomy. This rights-based obligation is operationally supported by Law no. 17 of 2023 on Health, which strengthens the role of primary healthcare services and community-based interventions while encouraging adaptive strategies responsive to local sociocultural contexts. Consequently, effective stunting prevention in indigenous settings such as the Baduy community requires institutionalized collaboration between traditional leaders and frontline health workers (bidan and kader posyandu), where biomedical practices are reframed within culturally acceptable meanings rather than imposed as external norms. Respecting cultural autonomy while strategically transforming symbolic practices into health-promoting behaviors is not only consistent with sociological theories of cultural capital but is also fully aligned with Indonesia’s current legal and policy architecture, making it a legally grounded and socially sustainable pathway for reducing stunting among indigenous populations.

Gender roles in Baduy society create a highly structured and segmented system of capital and authority that directly shapes nutritional outcomes. The mother’s role as primary caregiver constitutes a form of embodied cultural capital, encompassing her mastery of traditional feeding practices, herbal remedies, and daily child-rearing. This domestic-centered habitus means interventions like Posyandu sessions, which successfully transfer biomedical capital to mothers, can improve her knowledge and techniques. However, her agency is often circumscribed by a patrilineal resource field. The father, whose habitus is structured around provisioning and public representation, holds authority over the household’s economic capital—deciding what foods to purchase (e.g., choosing between fresh fish or cheaper salted fish) and when to allocate funds for clinic visits. Furthermore, in serious illness, the decision to escalate care from traditional herbs to a health center frequently requires deliberation with the father and often the paternal grandfather, reflecting a collective, male-dominated decision-making structure within the familial field. Thus, a mother may know a child needs nutrient-dense food or clinical care, but converting that knowledge into action depends on negotiating with male gatekeepers who operate on a different logic of resource allocation and risk assessment.

Consequently, effective nutrition interventions must adopt a dual-pathway strategy that empowers female caregivers while strategically engaging male authority structures. Empowering mothers and, crucially, grandmothers—who possess significant intergenerational symbolic capital as respected elders and repositories of traditional knowledge—as agents of change is essential. Programs should formalize their roles, for instance, by creating intergenerational “health circles” where grandmothers and mothers jointly adapt traditional recipes for nutritional enhancement, thereby blending cultural legitimacy with new information. Simultaneously, interventions must directly address the field of male authority. This involves framing nutritional investments not as a domestic expense but as contributing to the familial and communal capital valued by men: raising strong children who can uphold Baduy customs, contribute to farm labor, and ensure the lineage’s continuity. Engaging fathers and community leaders (Jaro) in dialogues that connect child nutrition to community resilience and cultural preservation can help convert health objectives into a form of symbolic capital recognizable within the male-dominated public sphere, thereby aligning household decision-making across gendered fields of habitus and power.

**Figure 11:**
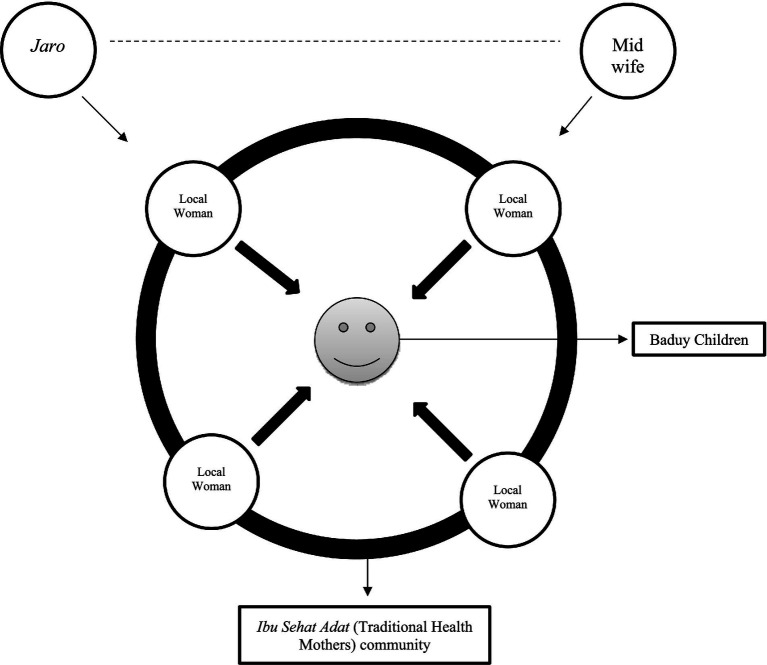
The multi-parties collaboration strategies diagram.

In relevance to the stronger legal framework, based on the data analysis, the woman can act as the community caregiver. According to the data, many of the women have more time than a man who has to go to the ladang, and they tend to be more inquisitive to the midwife. To strategically empower Baduy women as nutritional caregivers, a culturally embedded approach must convert their existing gendered cultural capital, rooted in traditional food knowledge, childcare, and gardening—into enhanced biomedical and symbolic capital through targeted, field-sensitive interventions. This involves establishing women-led garden learning circles where nutrition education integrates traditional ingredients like cassava, taro, and local greens with scientifically informed preparation methods—reframed not as replacement but as enhancement of customary practices. Training should be delivered in women-only, culturally familiar spaces such as Posyandu sessions or riverbank gatherings, using Sundanese oral traditions and participatory methods like recipe adaptation and intergenerational storytelling, while simultaneously creating bridging recognition systems that certify women both as modern health ambassadors and as Ibu Sehat Adat (Traditional Health Mothers) endorsed by customary leaders. To ensure sustainability, women should be equipped with portable nutrition kits and linked to economic ventures such as producing nutritious traditional snacks for tourists or medicinal herb kits, thereby transforming caregiving from a domestic role into a valued form of community leadership that operates within—and strengthens—the existing social and symbolic structures of Baduy life (Figure 11).

Later, intervention practices through Bourdieu’s concept of *field* highlight how effective stunting prevention in the Baduy community depends on negotiated collaboration among multiple social actors (Bourdieu, 2018). Field observations indicate that midwives (*bidan*) are able to engage more successfully with families when their activities are mediated by traditional leaders (*jaro*), who hold dominant symbolic capital within the customary field. For example, health education sessions related to maternal nutrition and child feeding were more accepted when delivered during customary gatherings or after obtaining explicit approval from *jaro*, rather than through standalone biomedical outreach. NGOs operating in the area also play a bridging role by translating biomedical objectives into culturally resonant messages, such as framing balanced nutrition as a means of maintaining bodily harmony rather than preventing disease. Families, particularly mothers and grandmothers, act as key agents in implementing these practices at the household level, yet their actions remain embedded in collective decision-making structures. These interactions illustrate that interventions function within a contested field where biomedical authority, customary leadership, and household practices intersect. Programs that recognize and strategically align these actors—rather than privileging a single form of authority—are more likely to gain legitimacy, sustain participation, and produce meaningful behavioral change.

## 6 Conclusion

In conclusion, this study highlights that stunting among the Baduy indigenous community cannot be understood solely through biomedical or infrastructural lenses but must be interpreted within the broader cultural, social, and symbolic frameworks that shape health behaviors and intervention acceptance. Despite overall national progress in reducing stunting prevalence, the Baduy case illustrates how entrenched cultural practices, intergenerational parenting patterns, food taboos, and community-level distrust of external health actors persist as significant barriers to effective intervention. By applying Bourdieu’s theoretical concepts of cultural capital and habitus, this research demonstrates how deeply rooted traditions and local logics of health create resistance to state-led programs, ultimately perpetuating disparities. These findings underscore the urgent need for culturally sensitive approaches that go beyond technical solutions and instead integrate local wisdom, engage traditional leaders, and build trust between health actors and indigenous communities. Addressing stunting in the Baduy requires not only health sector interventions but also broader social strategies that respect indigenous autonomy while ensuring children’s right to adequate nutrition and growth. As such, this study not only contributes to filling the gap in sociological research on indigenous health in Indonesia but also offers important insights for policymakers, development practitioners, and scholars concerned with designing inclusive, community-centered interventions that bridge the divide between modern health systems and traditional worldviews.

The findings of this study also highlight the need for further research on the effectiveness of traditional and community-based media in promoting behavioral change within indigenous contexts. In the Baduy community, information is primarily transmitted through oral traditions, customary gatherings, and messages delivered by respected elders rather than through written or digital media. These traditional communication channels carry high symbolic legitimacy and are closely aligned with local habitus and cultural capital, suggesting they may be more effective in influencing health-related behaviors than conventional public health messaging. However, empirical evidence on how such traditional media can be systematically integrated into nutrition and stunting prevention interventions remains limited. Future research should therefore examine the mechanisms, reach, and sustainability of culturally embedded communication strategies, as well as their potential to complement biomedical approaches. Understanding how traditional media can facilitate behavioral change while respecting cultural autonomy would provide valuable insights for designing more effective, context-sensitive health interventions in indigenous communities.

## Data Availability

The raw data supporting the conclusions of this article will be made available by the authors, without undue reservation.
